# Lactational High Fat Diet in Mice Causes Insulin Resistance and NAFLD in Male Offspring Which Is Partially Rescued by Maternal Metformin Treatment

**DOI:** 10.3389/fnut.2021.759690

**Published:** 2021-12-15

**Authors:** Hannah Hafner, Molly C. Mulcahy, Zach Carlson, Phillip Hartley, Haijing Sun, Maria Westerhoff, Nathan Qi, Dave Bridges, Brigid Gregg

**Affiliations:** ^1^Division of Endocrinology, Department of Pediatrics, Michigan Medicine, Ann Arbor, MI, United States; ^2^Department of Nutritional Sciences, School of Public Health, University of Michigan, Ann Arbor, MI, United States; ^3^Department of Pathology, Michigan Medicine, Ann Arbor, MI, United States; ^4^Department of Molecular and Integrative Physiology, Michigan Medicine, Ann Arbor, MI, United States

**Keywords:** lactation, dietary fat, developmental programming, NAFLD, metformin

## Abstract

Maternal metabolic disease and diet during pregnancy and lactation have important implications for the programming of offspring metabolic disease. In addition, high-fat diets during pregnancy and lactation can predispose the offspring to non-alcoholic fatty liver disease (NAFLD), a rising health threat in the U.S. We developed a model of maternal high-fat feeding exclusively during the lactation period. We previously showed that offspring from dams, given lactational high-fat diet (HFD), are predisposed to obesity, glucose intolerance, and inflammation. In separate experiments, we also showed that lactational metformin treatment can decrease offspring metabolic risk. The purpose of these studies was to understand the programming implications of lactational HFD on offspring metabolic liver disease risk. Dams were fed a 60% lard-based HFD from the day of delivery through the 21-day lactation period. A subset of dams was also given metformin as a co-treatment. Starting at weaning, the offspring were fed normal fat diet until 3 months of age; at which point, a subset was challenged with an additional HFD stressor. Lactational HFD led male offspring to develop hepatic insulin resistance. The post-weaning HFD challenge led male offspring to progress to NAFLD with more severe outcomes in the lactational HFD-challenged offspring. Co-administration of metformin to lactating dams on HFD partially rescued the offspring liver metabolic defects in males. Lactational HFD or post-weaning HFD had no impact on female offspring who maintained a normal insulin sensitivity and liver phenotype. These findings indicate that HFD, during the lactation period, programs the adult offspring to NAFLD risk in a sexually dimorphic manner. In addition, early life intervention with metformin via maternal exposure may prevent some of the liver programming caused by maternal HFD.

## Introduction

The field of developmental programming has provided irrefutable evidence that maternal exposures shape the metabolic health of the offspring for a lifetime (1, 2). Exposures during critical windows of development can permanently alter maturing offspring tissues, leading to increased susceptibility to metabolic decompensation. The lactation period is one critical susceptibility window; during which, alterations in milk composition can permanently change ongoing organ development and maturation, thus altering offspring organ function (3). Both maternal diet and maternal metabolic health during the lactation period play a role in transmitting this risk of metabolic syndrome to offspring (4, 5).

Previous studies have used cross fostering techniques and maternal diet isolated to the lactation period to show that, even in the absence of maternal obesity, diet composition has an ability to shape offspring metabolic outcomes (4, 6, 7). We and others have shown that feeding mouse dams a 60% high-fat diet (HFD) exclusively during the lactation period predisposes their offspring to obesity, insulin resistance, and glucose intolerance (4, 8). This programming is sexually dimorphic in rodents with males experiencing increased metabolic consequences and female offspring being relatively spared.

Non-alcoholic fatty liver disease marked by hepatic steatosis may progress from simple steatosis to non-alcoholic steatohepatitis (NASH), which is expected to become the leading cause of liver transplantation in the future (9). Dietary exposures play a role in the pathogenesis and progression of this liver disease. Overnutrition promotes obesity and insulin resistance, stimulating the release of fatty acids from adipose tissue into the blood and, eventually, the liver (10). In the process of *de novo* lipogenesis, carbohydrates are converted to free fatty acids in the liver by enzymes, such as SREBP1c, Acetyl CoA Carboxylase (ACC), and Fatty Acid Synthase (FAS). Overconsumption of carbohydrates and fat may lead to the accumulation of free fatty acids in the liver, and excess free fatty acids generate lipotoxic products, followed by a vicious cycle of hepatocellular injury, inflammation, and repair that can lead to fibrosis and hepatocellular carcinoma (10). With the rising prevalence of non-alcoholic fatty liver disease (NAFLD) and NASH (11), it is important to understand how life stages such as the lactation period can both contribute to the risk of lifetime metabolic liver disease and be targeted for intervention.

Lactational programming studies show that the neonatal liver is vulnerable to stressors. In response to a variety of stressors, offspring livers can be compromised. High carbohydrate diets fed to pups by gastrostomy, for example, led to increased adult liver fat (12). Lactational HFD also led to increased liver triglycerides and steatosis in several models (13–15). Small litters used to model early post-natal overnutrition show increased liver oxidative stress, with microsteatosis followed by insulin resistance (16, 17). The relationships between liver dysfunction and offspring insulin resistance in these models have not been evaluated in detail.

The objective of the current study is to dissect out the contributors to the insulin-resistant phenotype we detected in male offspring in previous studies. We used hyperinsulinemic, euglycemic clamps to understand the different tissue contributions to the whole-body phenotype. We also designed an intervention study to understand if the use of metformin during lactation, while the dam is consuming an HFD, could rescue the offspring metabolic programming. Metformin is the most commonly used oral anti-diabetes medication and can lower circulating insulin levels (18). It has been studied during the critical windows of pregnancy and lactation with some evidence for improved offspring health, following this exposure, although the results vary, depending on maternal health and dosing strategy (19–23). The lactation period is an underutilized window for intervention, and pharmacologic interventions may be more readily adopted by mothers than making a change in dietary fat consumption.

## Materials and Methods

### Animals

Mice were housed in ventilated cages in a facility with a 12-h light/dark cycle and provided with *ad libitum* access to food and water. All animal procedures were approved by the University of Michigan Institutional Animal Care and Use Committee. Two-month-old virgin C57BL/6J mice were purchased from Jackson Laboratories (Bar Harbor, ME, USA) and allowed to acclimate for 2 weeks prior to breeding. Upon parturition, a subset of dams was switched onto a 60% HFD (D12492 Research Diets New Brunswick, NJ, USA); remaining dams were maintained on 5001 (Normal Diet/ND) (13.5% kCal from fat, LabDiet, St. Louis, MO, USA) for the duration of the lactation period. For metformin rescue experiments, dams receiving HFD after parturition were also given sterile water bottles containing 3 mg/ml metformin-HCl (Spectrum Chemical, New Brunswick, NJ, USA). Bottles were changed weekly up to post-natal day 21. On post-natal day 21, offspring were weaned onto normal diet (ND). At 3 months of age, half of the offspring from the Ctrl or HFD dams were placed onto HFD for an additional 3 months. Experimental offspring generated fell into five groups: maternal normal diet (Ctrl PN), maternal normal diet with adult high-fat diet rechallenge (Ctrl PN + HFD), maternal high-fat diet (HFD PN), maternal high-fat diet with adult high-fat diet rechallenge (HFD PN + HFD), and maternal high-fat diet plus metformin (HFD + Met PN). Only offspring from litters with five to nine pups were used for the experimental groups, as this represents an average litter size in our colony. All experiments on adult offspring, with the exception of the hyperinsulinemic, euglycemic clamp, were performed on multiple, independent cohorts to demonstrate reproducibility. In one cohort using the same groups, non-fasted offspring were euthanized via CO_2_ inhalation at post-natal day 16 for blood and tissue collection. All other offspring were euthanized via CO_2_ inhalation at 6 months of age, and liver samples were either fixed in 4% formaldehyde or snap frozen and stored at −80°C for downstream analysis.

### Circulating Insulin Levels

At 2 months of age, offspring were fasted for 6 h, and blood was collected by tail snip and centrifuged to separate the serum. Blood from non-fasted, 16-day-old offspring was collected by cardiac puncture and centrifuged to separate the serum. Serum samples were analyzed for insulin concentration using a Mouse Ultrasensitive Insulin ELISA (ALPCO, Salem, NH, USA). The sensitivity for this test is 0.115–6.9 ng/ml with a reported coefficient of variation of <9.3%.

### Insulin Tolerance Test

Mice were subjected to insulin tolerance tests at 5 months of age (Ctrl PN *n* = 6, HFD PN *n* = 15, HFD + Met PN *n* = 9). Mice were fasted in the morning for 6 h prior to testing. Each mouse was administered 0.75 units/kg of insulin lispro via intraperitoneal injection. Blood glucose was measured after fasting, prior to injection, and at 15, 30, 60, and 90 min after injection using a Bayer Contour glucometer (Bayer AG, Leverkusen, Germany). The area under the curve was calculated for each animal as the sum of glucose values during the experiment. To test initial responsiveness to insulin administration, we calculated individual rates of fall by limiting the data to the first 45 min of the experiment, and then regressing log glucose by time for each animal. This resulted in an individual rate and intercept for each animal. Slopes were then calculated by multiplying the exponentiated intercept by the rate, generating rate estimates in mg/dl.

### Hyperinsulinemic-Euglycemic Clamp

The hyperinsulinemic-euglycemic clamp (HEC) study was carried out by the University of Michigan Animal Phenotyping Core. Male Ctrl PN (*n* = 9), HFD PN (*n* = 12), and HFD + Met PN (*n* = 11) offspring aged 3 months exposed to either HFD or ND during the lactation period underwent surgical implantation of indwelling catheters in the right carotid artery and right jugular vein. The catheters were subcutaneously tunneled and exteriorized at the back of the neck. After recovering from implantation surgery (5 days), conscious and unrestrained animals underwent a 5-h fast before initiation of the clamp study. A priming dose of [3-H^3^] glucose (1 μCi) was administered beginning at 90 min before insulin administration (*t* = −90 min), followed by infusion of .05 μCi per minute for 90 min. The insulin clamp began at *t* = 0 with a prime-continuous infusion (16 mU/kg bolus, followed by 4.0 mU/kg/min) of human insulin (Novo Nordisk). The infusion of [3-H^3^] glucose was increased to 0.10 μCi/min for the rest of the experiment. Blood samples were collected via the carotid artery at *t* = −10 and 120 (for basal and final insulin) as well as 80, 85, 90, 100, 110, and 120 min for glucose specific activity.

Continuous glucose infusion (50% glucose in phosphate-buffered saline) began at time = 0 min and continued throughout the 120-min procedure. Blood glucose was monitored with a glucometer (Accu-check, Roche, Basel, Switzerland) every 10 min, and adjustments were made to the glucose infusion rate for each animal to remain euglycemic (120–130 mg/dl). To determine tissue-specific uptake of glucose, a bolus injection of [1-14C]-2 deoxyglucose (10 μCi) was administered at *t* = 78 min. At the end of the clamp (*t* = 120), animals were anesthetized with an intravenous infusion of sodium pentobarbital. The liver, heart, gastrocnemius, brown adipose depot, inguinal white adipose depot (subcutaneous), and epidydimal adipose depot (visceral) were collected, weighed, and snap-frozen in liquid nitrogen and kept at −80°C for later use. [14C]2-Deoxyglucose-6-phosphate was then determined from tissues using a liquid scintillation counter. Hepatic tissue samples collected during the HEC were used for determination of *de novo* lipogenesis (DNL) by calculating disintegrations per minute per mg of lipid extracted from the liver tissue. Serum non-esterified fatty acid (NEFA) levels were determined from samples collected before and during the clamp study using the NEFA-HR (2) kit (Catalog No. 276-76491; Wako Diagnostics) in accordance with manufacturer's guidelines. Percent suppression of NEFA and EGP was calculated by subtracting the value for either measure during the clamped portion of the experiment from the basal value, and then dividing by the basal value and multiplying by 100.

### Picro-Sirius Red Staining

Picro-sirius red staining (also called Sirius red staining) highlights fibrosis by staining collagen (Collagen I and III fibers) fibers in formaldehyde-fixed, paraffin-embedded liver tissue sections (24). Sirius red-stained collagen appears red by light microscopy. Tissue sections were deparaffinized and rehydrated followed by staining in Picro-sirius Red solution for 1 h. Slides were dehydrated in three changes of 100% ethanol, followed by xylene and mounted with Permount medium. The images were taken on an Olympus microscope (Olympus Corporation of the Americas, Center Valley, PA, USA) and analyzed using freely available Image J software. Five sections were examined for each animal.

### Hematoxylin and Eosin Staining

Formaldehyde-fixed, paraffin sections of liver were cut at 5 μm. All tissues were stained as described previously (20).

### Immunofluorescent Staining

Formaldehyde-fixed, paraffin embedded liver samples were sectioned at 6 μm. After deparaffinization and antigen retrieval, the slides were blocked and permeabilized with 5% BSA PBS with 0.1% Triton × 100 for 20 min at room temperature. The slides were incubated with a primary antibody at 4°C overnight. Primary antibodies used were polyclonal anti-caveolin (Cell Signaling Technology Cat # 3238 Danvers, MA, USA) and monoclonal anti-Mac2 (Galectin 3, eBioscience M3/38) (Thermo Fisher Scientific, San Diego, CA, USA). This was followed by a secondary antibody, fluorescein isothiocyanate anti-rabbit (1:100; Jackson ImmunoResearch Laboratories, West Grove, PA, USA). Slides were mounted with a Vectashield mounting medium containing DAPI mounting media. The sections were imaged on an Olympus microscope.

### Hepatic Triglyceride Quantification

The total triglycerides in liver were analyzed by Thermo Scientific™ Triglycerides Reagent (TR22421), following the manufacturer protocol. The triglycerides in liver were extracted using the previously described protocol (25). The triglyceride results were normalized to the mass of the liver tissue initially used for assay, as previously reported (26).

### Microarray Methods

For RNA microarray studies, an Affymetrix Plus WT GeneAtlas2.1 ST array platform was used. Biotinylated cDNA was prepared according to the Affymetrix Plus WT kit protocol (GeneChip^®^ WT Plus Reagent Kit Manual P/N 703174 Rev. 2) from 400 ng total RNA. Following the labeling procedure, 2.76 ug of cDNA was hybridized at 48°C on Mouse Gene 2.1 ST and was washed and stained using the Affymetrix Gene Atlas system (software version 2.0.0.460). Peg Arrays were scanned using the Affymetrix Gene Atlas system (software version 2.0.0.460). RMA was used to fit log2 expression values to the data using the oligobioconductor package in R version 3.4.3. For gene set enrichment analysis, transcript-level data were reduced to gene-level data via tximeta (27) and txiimport (28) prior to analysis by DESeq2 (29) v1.28.1. For gene set enrichment analyses, we used ClusterProfiler v3.16 after conversion of mouse genes to human genes and ranking by fold change (30, 31). Data are available from GEO at accession No. GSE182071.

### RNA Extraction and qRT-PCR

Ribonucleic acid was extracted from frozen liver samples using an RNeasy Mini Kit (QIAGEN, Gaithersburg, MD, USA) according to the manufacturer's instructions. cDNA was generated using a high-capacity cDNA reverse transcription kit (Life Technologies, Carlsbad, CA, USA), followed by analysis using real-time PCR with Power SYBR Green PCR Master Mix (Applied Biosystems, Grand Island, NY, USA). The gene *Gapdh* was used as an internal control. A list of primer sequences can be seen in Table 1.

**Table 1:** Sequences for qPCR primers.

**Gene**	**Forward 5^**′**^-3^**′**^**	**Reverse 5^**′**^-3^**′**^**	**NCMI ID**
*Angptl4*	GGA CCT TAA CTG TGC CAA GA	CGT GGG ATA GAG TGG AAG TAT TG	
*Cyp4a14*	TCT CAT CTT TCT GCC CTC ATT TC	CAG TGG CTG GTC AGA GTT AAA G	
*Cyp7a1*	CAC CTT GAG GAT GGT TCC TAT AA	TCA AAG GGT CTG GGT AGA TTT C	
*Fabp4*	GTG AAG AGC ATC ATA ACC CTA GAT	CAC GCC TTT CAT AAC ACA TTC C	
*Fbp1*	TTC ACC GCA CTC TGG TAT ATG	CGG CCT TCT CCA TGA CAT AA	NM_09395.3
*Foxo1*	CGT GCC CTA CTT CAA GGA TAA G	GCA CTC GAA TAA ACT TGC TGT G	
*G6pc*	CAA CAG CTC CGT GCC TAT AA	TAG CAA GAG TAG AAG TGA CCA TAA C	NM_008061.4
*Gapdh*	AAC AGC AAC TCC CAC TCT TC	CCT GTT GCT GTA GCC GTA TT	GU214026.1
*Pck1*	GGC ACC TCA GTG AAG ACA AA	CGA TGA CTT CCC AGT AAA CA	NM_011044.3
*Plin2*	GAA GGA TGT GGT GAC GAC TAC	TCA CTG CTC CTT TGG TCT TAT C	
*Ppara*	CGG TGT GTA TGA AGC CAT CT	TAA GGA ACT CGC GTG TGA TAA A	
*Zbtb16*	CGT CTG TGG ATC TGA ACT GTA TC	AGG AAG GAA GGA AGG AAG GA	

*NCMI IDs are listed when available*.

### Western Blotting

Liver tissue collected at the end of the hyperinsulinemic-euglycemic clamp was snap frozen in liquid nitrogen and stored at −80°C. Liver tissue was cut on dry ice and homogenized in a RIPA buffer. Samples were run on SDS-PAGE gel and transferred overnight onto nitrocellulose membranes. Total protein was assessed via REVERT stain (Li-COR Biosciences, cat # 926-11011) and was used to normalize target signals [AKT and phosphor-AKT(ser473)]. Nitrocellulose membranes were then blocked in 2% Bovine Serum Albumin for 1 h before being incubated with a primary antibody. Primary antibodies used were P-AKT: Cell Signaling Technology, Catalog # 4060S (Danvers, MA, USA) (rabbit) and AKT (pan): Cell Signaling Technology, Catalog # 2920S (mouse) (Danvers, MA, USA). Blot was then washed in TBST before incubation with a secondary antibody. Blots were imaged using Li-COR scanner and quantified using Image Studio. All protein targets were normalized to total protein.

### Statistical Analysis

#### Insulin Tolerance Test

Statistical analyses for insulin tolerance testing were completed using mixed linear effects models, with fixed effects of time and experimental treatment, and random effects of individual mouse ID and maternal mouse ID. Further analysis on the initial rate of drop in response to insulin administration was conducted by limiting the dataset to observations before 20 min. Then, linear models were constructed with effect of the group and of time and the interaction thereof. The coefficient of the interaction estimate was then evaluated. Models with *p* < 0.05 were considered to be statistically significant.

#### Hyperinsulinemic Euglycemic Clamp

The assumption of euglycemia was assessed via mixed linear modeling and was found to be similar between all control and HFD groups. Hyperinsulinemia assumption was tested by two-way ANOVA. Analysis of glucose infusion rate (GIR) was conducted by use of mixed linear effects modeling, with fixed effects of time and experimental group and random effect of individual animal IDs. The area under the curve was determined as the sum of GIR values for the duration of the experiment and was assessed via pairwise testing. Values were assessed for normality using Shapiro–Wilk tests and equivalence of variance using Levene's test. When values failed tests of normality, non-parametric tests were used instead of Student's *T*-test.

#### Other Results

Data are shown as mean ± standard error of the mean. Data were checked for normality and equal variance using the Shapiro–Wilk and Brown–Forsythe tests. Two-tailed *T*-tests, Welch's Student's *T*-test or Mann–Whitney *U*-tests were used as appropriate to compare results from two groups. In the metformin experiments where three groups were analyzed, one-way ANOVA was performed to analyze for differences between treatment groups, followed by pairwise *T*-tests. Significance was determined as *p* < 0.05. Statistical analyses were performed using GraphPad Prism 9.0 software.

## Results

### An Offspring Metabolic Profile After Lactational Exposure to HFD

**Figure 1:**
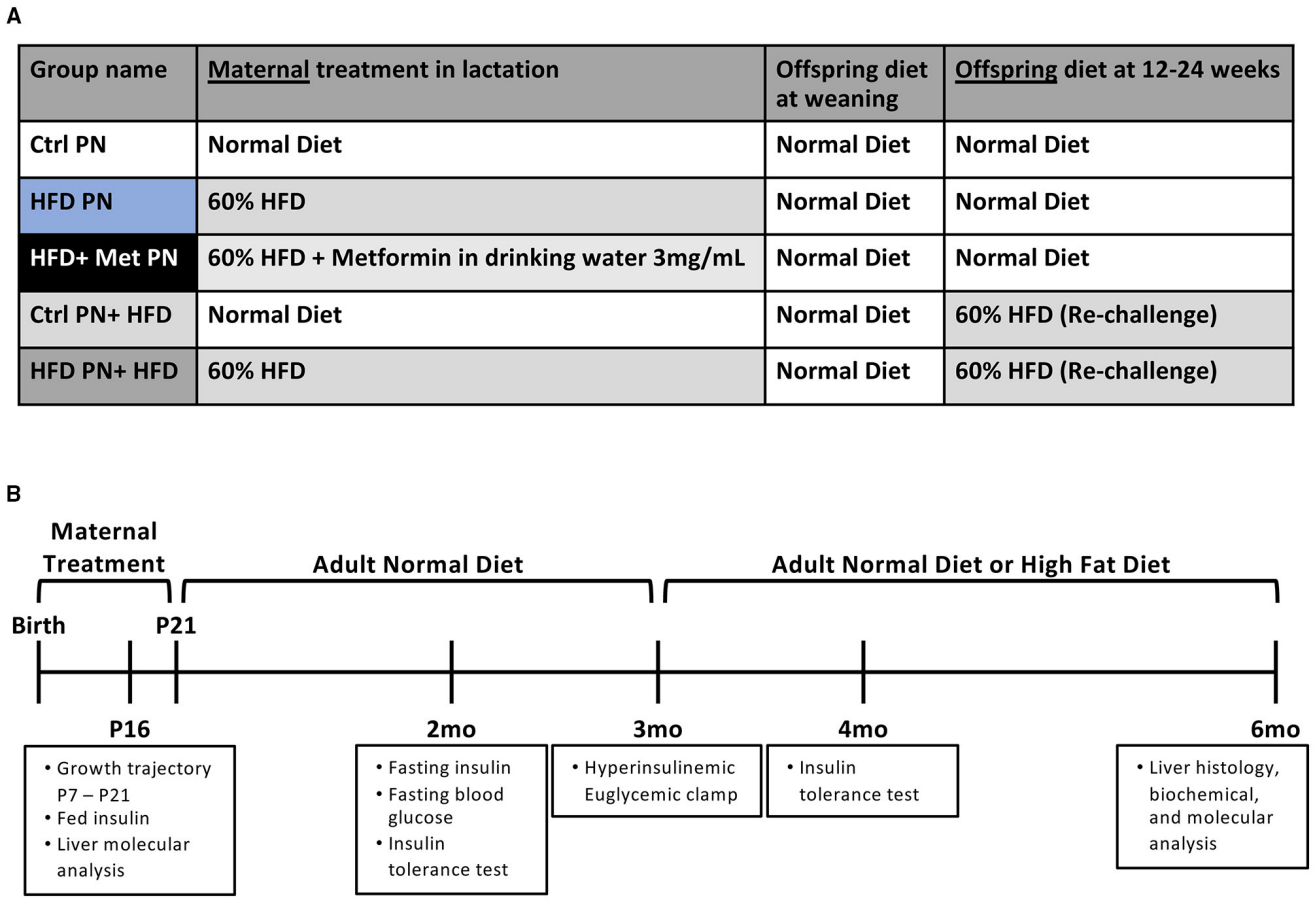
Experimental schema for the experiments presented. **(A)** A scheme of diet conditions per group. **(B)** Experimental timeline. Ctrl, control; HFD, high-fat diet; PN, post-natal; Met, metformin.

**Figure 2:**
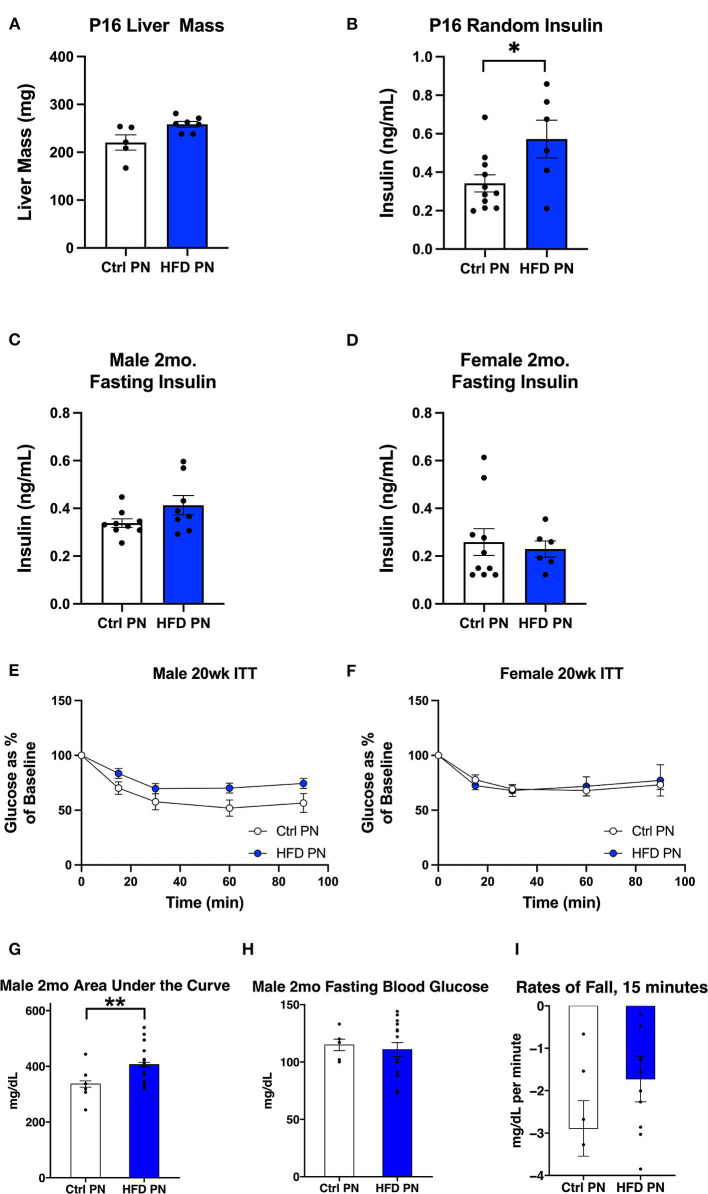
Offspring metabolic characteristics after lactational exposures. All data are presented as a litter average with the n reflecting the number of litters studied. All white values are for Ctrl PN and blue are for HFD PN offspring. **(A)** Post-natal day 16 (P16) Liver Mass **(B)** P16 Fed Insulin **(C)**. Fasting insulin at 2 months in male offspring **(D)**. Fasting insulin at 2 months in female offspring **(E)**. Insulin tolerance test at 5 months in male offspring (*n* = 6–7 litters) **(F)**. Insulin tolerance test at 5 months in female offspring (*n* = 4–7 litters) **(G–I)** constitute further analysis of the 5-month male ITT **(G)**. The area under the curve from 100% for the ITT glucose values **(H)**. Fasting blood glucose at the start of the ITT **(I)**. The rate of fall in blood glucose from the baseline to 15 min during the test. Ctrl, control; HFD, high-fat diet; PN, post-natal. *p < 0.05.

The experimental groups timeline are shown in Figures 1A,B. We first examined offspring insulin sensitivity during and after the lactational HFD exposure. The groups we examined were offspring of dams-fed ND during lactation (Ctrl PN) and offspring of dams-fed HFD during lactation (HFD PN). Male and female data from birth to weaning were combined, as we do not observe sex differences until mice reach adulthood. As we and others have previously described, HFD PN offspring have an elevated circulating insulin level at post-natal day 16 (P16) (Figure 2B) and heavier weights during the lactation period on days 7, 14, and 21 (*p* < 0.0001 at each timepoint) (**Figure 6A**) (8, 32). Despite this, we did not detect a change in the offspring liver weights during the lactation period (Figure 2A). There was also no difference in circulating triglyceride levels between Ctrl PN and HFD PN pups at P16 (data not shown). Fasting insulin levels at 2 months were unchanged in both male and female HFD PN offspring fed ND (Figures 2C,D). Insulin tolerance testing was conducted in Ctrl PN and HFD PN offspring at 5 months of age (Figures 2E,F). Male HFD PN offspring had similar fasting blood glucose levels compared to control males (*p* = 0.6, Figure 2H) but had a 16% greater area under the curve after insulin administration, suggesting some level of insulin resistance in this group (*p* = 0.003, Figure 2G). However, mixed linear modeling failed to reach statistical significance (*p*_diet_ = 0.075). There was no difference between groups in the rate of fall in blood glucose in the first 15 min of the ITT (Figure 2I). Female offspring had no difference in insulin response between groups (Figure 2F).

### Hyperinsulinemic Euglycemic Clamp

**Figure 3:**
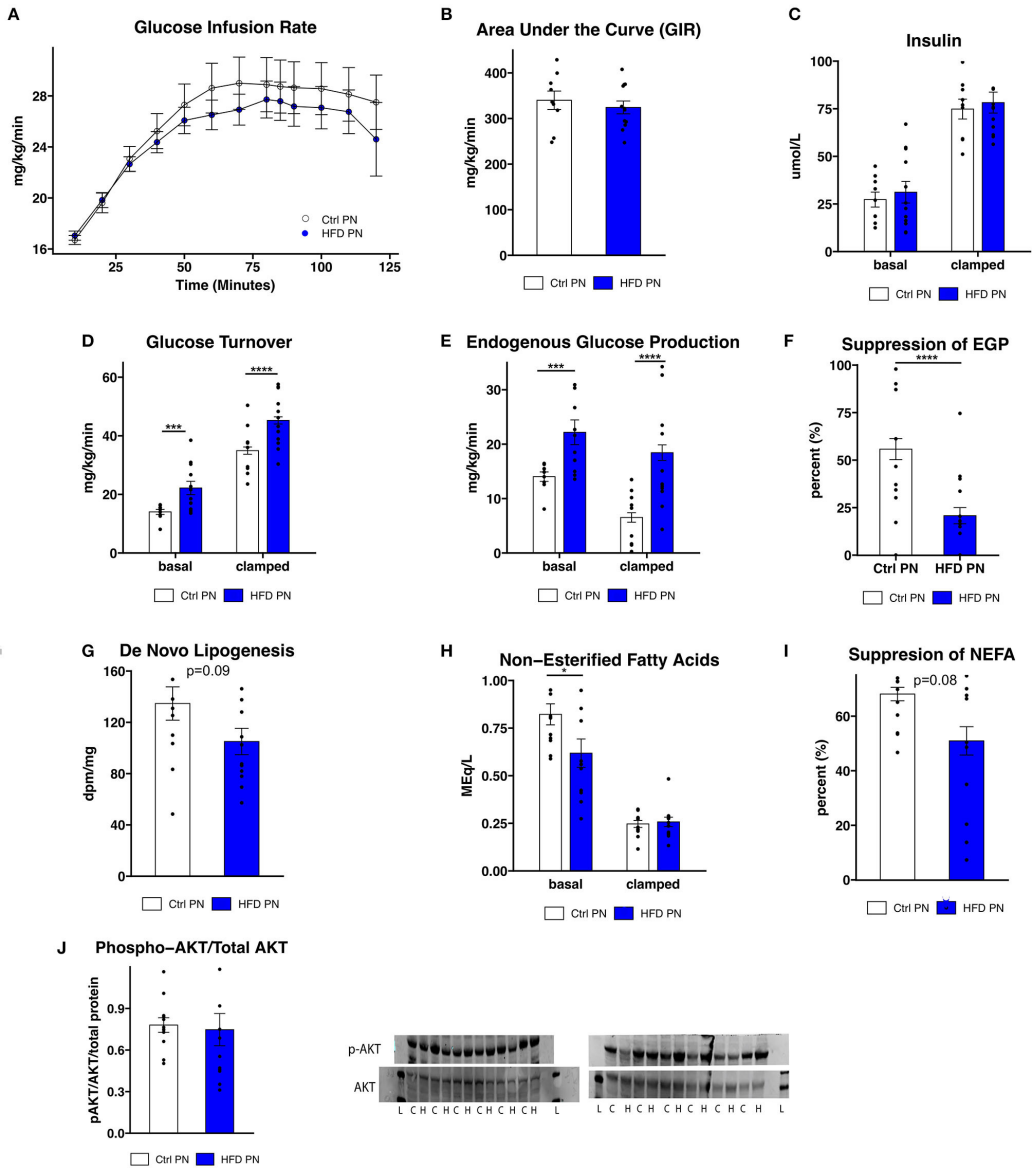
Insulin sensitivity and glucose production. Three-month-old male mice whose mothers either had normal diet (Ctrl PN, *n* = 9) or high-fat diet (HFD PN, *n* = 12) during the lactation period underwent a hyperinsulinemic euglycemic clamp, beginning after a 6-h fast. **(A)** Time course of glucose infusion rate (GIR) over the course of the clamp study. **(B)** The area under the curve for GIR **(C)**. Insulin levels at basal and clamped conditions **(D)**. Glucose turnover at basal and clamped conditions. **(E)** Endogenous glucose production (EGP) at basal and clamped conditions **(F)**. Percent suppression of EGP **(G)**. *De novo* lipogenesis in liver tissue in response to insulin **(H)**. Non-esterified fatty acids (NEFA) in serum at basal and clamped conditions **(I)**. Suppression of NEFA in serum **(J)**. Western blot of liver tissue showing AKT and p-AKT (ser473) protein levels. Western blot abbreviations: C, Ctrl PN; H, HFD PN; L, Ladder. Other abbreviations: Ctrl, control; HFD, high-fat diet; PN, post-natal. *p < 0.05, ***p < 0.001, ****p < 0.0001.

To further assess the differences in glucose homeostasis identified by ITT in male offspring, a hyperinsulinemic euglycemic clamp was conducted at 3 months of age in one cohort of male offspring. The glucose infusion rate (Figure 3A), representing whole body insulin sensitivity, was similar between Ctrl PN and HFD PN males, consistent with modest insulin resistance. The glucose infusion rate area under the curve (Figure 3B) did not differ between the Ctrl PN and the HFD PN group (*p* = 0.5), nor did insulin concentration differ between groups at basal (*p* = 0.6) or clamped (*p* = 0.7) conditions (Figure 3C). Insulin turnover was similar between groups. HFD PN males had 37% higher glucose turnover at basal conditions (*p* = 0.008) and 30% higher when clamped (*p* < 0.0001; Figure 3D). Thus, there is greater glucose disposal in HFD PN males under both basal and insulinemic conditions. Endogenous glucose production (EGP) was 58% higher in HFD PN males at basal (*p* = 0.008) and 183% higher in clamped conditions (*p* < 0.0001; Figure 3E). Consistent with this, we found a 63% greater overall suppression of EGP achieved during the clamp in Ctrl PN males (*p* < 0.0001; Figure 3F). This suggests that HFD PN males developed insulin resistance of the liver, with impaired suppression of hepatic glucose production in response to insulin. We also found that *de novo* lipogenesis in response to insulin in the liver of HFD PN males was 22% lower than Ctrl PN males without reaching statistical significance (*p* = 0.09, Figure 3G), possibly indicating mild hepatic insulin resistance. Non-esterified fatty acids (NEFA) were 25% lower in basal conditions for HFD PN males (*p* = 0.04, Figure 3H basal conditions), indicating lower levels of lipolysis. The total suppression of NEFAs in the hyperinsulinemic state was 25% lower in HFD PN males; however, this did not reach statistical significance (*p* = 0.08, Figure 3I). It is plausible that these baseline differences in the amount of lipolysis in HFD PN males drive increased hepatic gluconeogenesis. These differences may also not be specific to the effects of insulin as NEFA levels were similar between Ctrl PN and HFD PN males under hyperinsulinemic conditions (*p* = 0.99, Figure 3H clamped conditions). We next examined liver-specific insulin signaling through western blotting in liver tissue collected immediately after the clamp by assessing AKT and phosphorylated (serine 473) AKT (Figure 3J) as a major readout. We found no reduction in insulin signaling in the livers of HFD males (*p* = 0.4), suggesting the insulin resistance apparent from the clamp study is not fully attributable to liver-specific AKT phosphorylation. We anticipated that the increased peripheral glucose disposal in HFD PN males (Figure 3D) would be evident in tissue-specific uptake of glucose. When individual tissues were analyzed, however, three of the five tissues showed significantly lower glucose uptake in HFD males, 25% lower in heart (*p* = 0.003), 29% lower in subcutaneous adipose (*p* = 0.03), and 37% lower in visceral adipose (*p* = 0.02, Supplementary Figure 1). This suggests that glucose turnover may have been elevated in a tissue that was not collected for analysis, potentially brain or liver tissues. Our interpretation is that this is unrelated to insulin signaling as the glucose turnover was elevated in both basal and hyperinsulinemic conditions. Taken together, we present evidence of some degree of insulin resistance at the liver, heart, and adipose tissue of male HFD PN offspring. However, the differences in baseline measures between Ctrl PN and HFD PN males may implicate a non-insulin dependent mechanism or tissue in the development of this phenotype.

### Liver Pathology in Male High-Fat Diet Offspring

**Figure 4:**
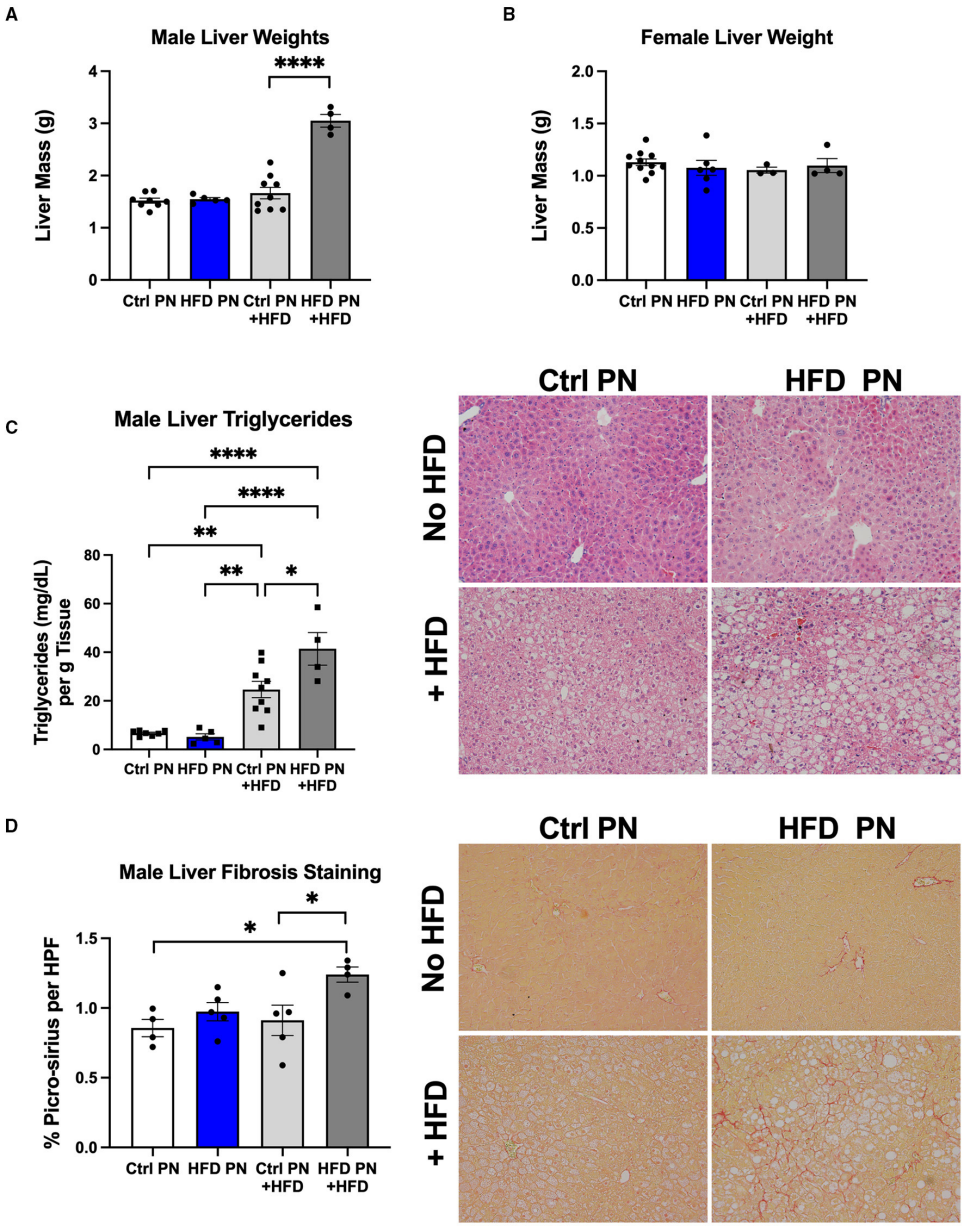
Offspring liver morphology and metabolic characteristics. Liver weights at necropsy (6 months of age) in male **(A)** and female **(B)** offspring during both ND feeding (no HFD) and HFD rechallenge. ND results are white for Ctrl PN and blue for HFD PN. HFD rechallenge results are in light gray for Ctrl PN + HFD and dark gray for HFD PN + HFD **(C)**. Liver triglyceride quantification and representative histology by hematoxylin and eosin (H&E) staining to visualize fat accumulation as open spaces. **(D)** Liver fibrosis quantification by picro-sirius staining with representative images showing red-stained collagen fibers. Ctrl, control; HFD, high-fat diet; PN, post-natal; HPF, high-power field. *p < 0.05, **p < 0.01, ****p < 0.0001.

Due to the evidence of physiological liver insulin resistance on the hyperinsulinemic euglycemic clamp, we next focused on analysis of the liver morphology. We examined the livers in two separate experimental conditions. We first studied livers of male and female Ctrl PN and HFD PN offspring at 6 months of age that had been weaned onto and maintained on ND throughout life. In the other condition, we rechallenged the offspring in both Ctrl PN and HFD PN groups with a “second-hit” stressor of an HFD from 3 to 6 months of age. This “second-hit” is sometimes necessary to unmask programmed metabolic defects. There was no difference in whole liver mass in either male or female mice maintained on ND. After HFD rechallenge, we observed a significant increase in whole liver mass in male HFD PN offspring, which was not seen in female offspring (Figures 4A,B). We continued our liver morphology investigation in male mice, given the lack of differences in liver mass and insulin resistance in female mice reported here and previously (8). We found that male offspring from both Ctrl PN and HFD PN maintained on ND had no evidence of liver fat accumulation or fibrosis (Figures 4C,D). Pathologist examination of hematoxylin and eosin-stained liver specimens (Supplementary Table 1) showed no abnormalities both at post-natal day 16 and 6 months of age in the Ctrl PN and HFD PN groups. Male HFD PN + HFD offspring developed evidence of hepatic steatosis and fibrosis. Pathologist review of male Ctrl PN + HFD offspring showed that 4/7 had evidence of microvesicular steatosis with 1/7 also showing macrovesicular steatosis. HFD PN + HFD male offspring showed that 5/5 had evidence of both microvesicular and macrovesicular steatosis. Hepatic triglyceride levels were elevated to a greater degree in male HFD PN + HFD offspring compared with Ctrl PN + HFD male offspring (Figure 4C). There was also an increased percentage of collagen fibers stained with picro-sirius red per high-powered field in male HFD PN + HFD offspring, indicating increased liver fibrosis in this group (Figure 4D). Taken together, our results show that maternal HFD feeding during lactation increases susceptibility of male mice to hepatic fibrosis and fat accumulation upon HFD rechallenge later in life.

### Hepatic Upregulation of Gluconeogenesis, the PPAR-Alpha Pathway, and Inflammation Genes

To further understand the molecular events underlying the liver pathology observed in offspring, we extracted RNA from 6-month adult male offspring liver in a non-fasted state and performed an RNA array using an Affymetrix platform to examine differences in transcriptomes between the Ctrl PN and HFD PN groups. When using an exploratory *p*-value cut-off of *p* = 0.01, there were 85 genes with increased expression and 18 genes with decreased expression in HFD PN males compared with Ctrl PN. When the results of this pilot gene expression study were analyzed using gene set enrichment analysis, the major enhanced pathway was the peroxisome proliferator-activated receptor (PPAR)-alpha pathway. Among the genes with the most elevated expression in HFD PN males were *Cyp4a14* and *Zbtb16*. Among those with the highest effect size for decreased relative expression was *Cyp7a1*. We then went on to perform individual qPCR on a larger set of samples to validate the array results and to examine similar gene candidates at other time points during the life course.

**Figure 5:**
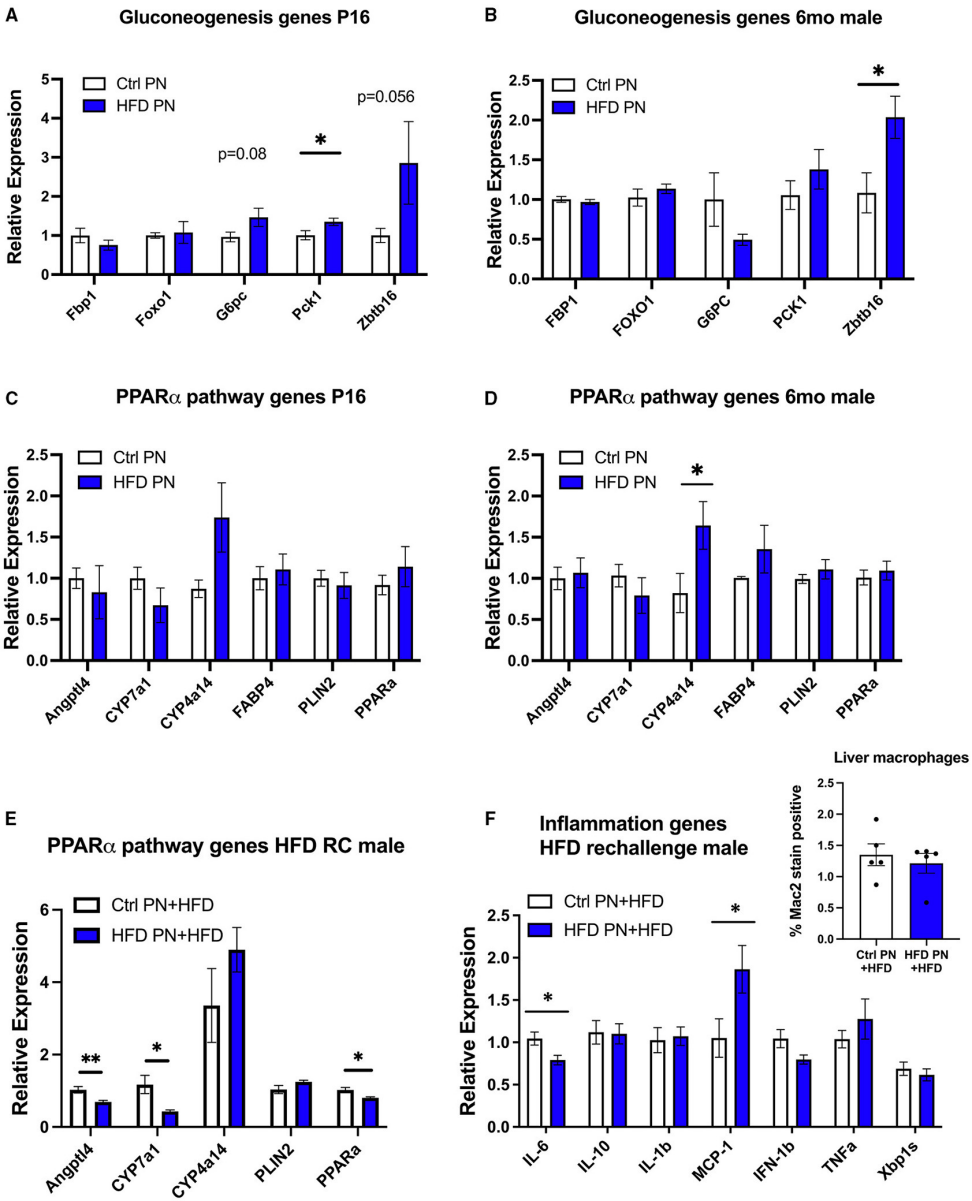
Offspring hepatic gene expression across the lifespan. Gene expression data are representative of individual mice and not averaged per litter. White values are for Ctrl PN, and blue are for HFD PN offspring. Gluconeogenesis genes at post-natal day 16 (P16) Ctrl PN *n* = 9–10, HFD PN *n* = 4–7 per gene **(A)** and in adult male offspring maintained on ND at 6-month Ctrl PN *n* = 4–5, HFD PN = 4–5 per gene **(B)**. PPAR-alpha pathway genes at P16 Ctrl PN *n* = 9–12, HFD PN *n* = 10–13 per gene **(C)**, and in adult male offspring maintained on ND at 6-month Ctrl PN *n* = 7–8, HFD PN *n* = 7–9 per gene **(D,E)**. PPAR-alpha pathway genes in male offspring rechallenged with HFD at 6-months Ctrl PN + HFD *n* = 8, HFD PN + HFD *n* = 8 per gene **(F)**. Inflammatory genes in male offspring rechallenged with HFD at 6-months Ctrl PN + HFD *n* = 6–8, HFD PN + HFD *n* = 8 per gene with quantitation of liver macrophages in HFD rechallenged males at 6 months using immunofluorescent staining for Mac2 (inset). Ctrl, control; HFD, high-fat diet; PN, post-natal. *p < 0.05, **p < 0.01.

Due to the changes in hepatic glucose production, we first analyzed genes related to hepatic gluconeogenesis. At P16, we detected a significant increase in the gluconeogenesis enzyme *Pck1* with a trend toward an increase in *G6pc* and *Zbtb16*, although this did not reach statistical significance (*p* = 0.08 and *p* = 0.056, respectively, Figure 5A). In adult male HFD PN liver, only *Zbtb16* had significantly increased expression (Figure 5B). Given the significant enrichment of PPAR-alpha pathway genes in our pilot array, we examined these candidates in P16 liver where there were no differences between groups that reached statistical significance (Figure 5C). In the adult male HFD PN offspring, we detected a significant increase in *Cyp4a14* (Figure 5D). In adult male HFD PN + HFD offspring, we detected some different changes in the PPAR-alpha pathway with decreased *Angptl4, Cyp7a1*, and *Ppara* (Figure 5E). Overall, *Cyp4a14* was highly expressed in both HFD rechallenge groups. We previously showed an increase of inflammatory macrophages in gonadal white adipose tissue in HFD PN + HFD male mice (8). Therefore, we examined expression of inflammatory genes and found elevated *Mcp-1* and decreased *Il-6* expression in HFD PN + HFD male livers (Figure 5F). When we examined the livers with Mac2 immunofluorescent staining to evaluate for the presence of macrophages, there was no clear difference between the Ctrl PN + HFD and HFD PN + HFD groups (Figure 5F, inset). Overall, we found an increase in *Zbtb16* and *Cyp4a14* expression in adult males that may be associated with lactational HFD exposure and could explain liver phenotypes detected in our model.

### Maternal Metformin Administration in Addition to HFD Counteracts Some Offspring Metabolic Outcomes

**Figure 6:**
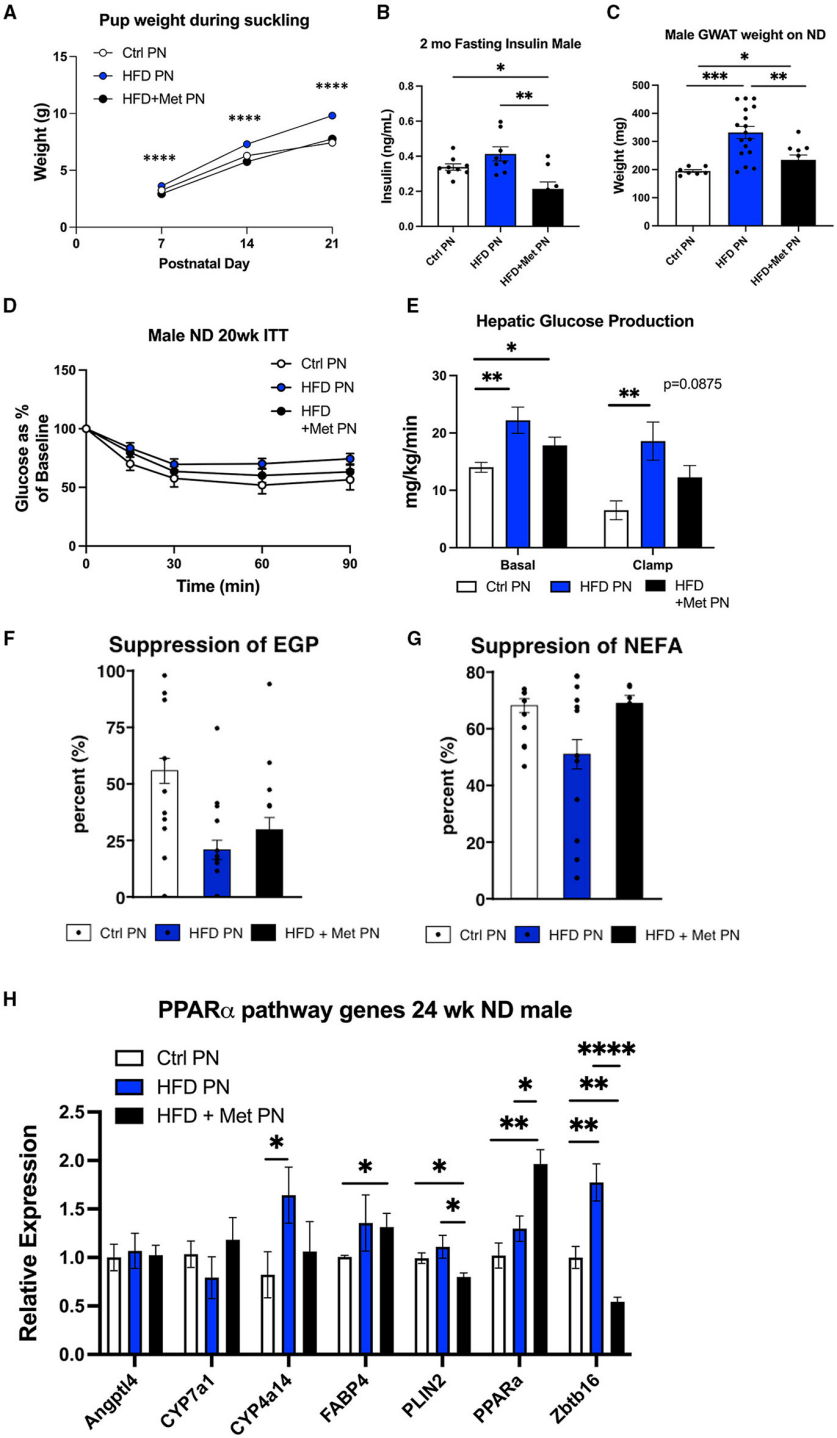
Impact of metformin given during lactation at the time of HFD exposure on offspring. White values are for Ctrl PN, blue are for HFD PN, and black are for HFD + Met PN offspring **(A)**. Pup weights during lactation averaged by litter (*n* = 6–7 litters) **(B)**. Fasting insulin in male offspring at 2 months of age **(C)**. Visceral fat pad weight (GWAT) in male offspring at necropsy at 6 months **(D)**. Insulin tolerance test at 5 months (*n* = 7–8) **(E)**. Hepatic glucose production at basal and clamped conditions during the hyperinsulin mice uglycemic clamp (HEC). This is the same clamp as in Figure 3 in which Ctrl PN and HFD PN results were shown, repeated here with the addition of the HFD + Met PN group (*n* = 8–11) **(F)**. Percent suppression of endogenous glucose production **(G)**. Percent suppression of lipolysis on clamp **(H)**. PPAR-alpha pathway genes in adult male offspring-fed ND at 6 months (*n* = 5–8). Ctrl PN and HFDPN expression results were previously shown in Figure 5D, repeated here with the addition of the HFD + Met PN group. Ctrl, control; HFD, high-fat diet; PN, post-natal; Met, metformin. *p < 0.05, **p < 0.01, ***p < 0.001, ****p < 0.0001.

We have previously demonstrated the ability of metformin exposure during lactation to program decreased offspring adiposity and protection from the effects of HFD rechallenge (20). We next wanted to understand the impact of metformin given to dams at the same time as lactational HFD on offspring metabolic outcomes. We observed that metformin returned the suckling offspring weights on days 7, 14, and 21 to the same as those of the Ctrl PN offspring (Figure 6A). HFD + Met PN offspring had lower-fasting insulin levels at 2 months of age than both Ctrl PN and HFD PN offspring (Figure 6B), and had decreased visceral adiposity at 6 months of age, as measured by GWAT weight (Figure 6C), compared to HFD PN males. During the 5-month ITT, the HFD + Met PN males were more responsive to insulin administration than the HFD PN males. However, HFD + Met PN males did not fully recapitulate the insulin sensitivity of Ctrl PN males (Figure 6D). Under hyperinsulinemic euglycemic clamp conditions, the suppression of hepatic glucose production of HFD + Met PN males did not differ from that of Ctrl PN or HFD PN groups (Figures 6E,F). Baseline NEFA levels tended to normalize in HFD + MetPN offspring with levels 35% higher than in HFD PN offspring (*p* = 0.078). Suppression of lipolysis also tended to improve in the HFD + MetPN group with suppression 35% higher than in HFD PN, although this did not reach significance (*p* = 0.10, Figure 6G). In terms of the key liver genes altered by lactational HFD, metformin increased expression of *Ppara* along with decreasing *Plin2* and *Zbtb16* (Figure 6H).

## Discussion

In these experiments, we demonstrated that a brief exposure of dams to HFD during lactation has lifelong implications for the offspring and that concurrent maternal metformin exposure may provide some protection in adulthood. Metabolic abnormalities were detected in HFD PN offspring on the normal diet, which included liver and adipose tissue insulin resistance. A “second-hit” stressor of HFD in adult life caused progression to liver steatosis to a greater extent in HFD PN offspring, and fibrosis which was not observed in Ctrl PN offspring. When metformin was given to dams being fed HFD, some of the metabolic phenotypes in male offspring were rescued along with a lower-fasted insulin level and a trend toward improved liver sensitivity to insulin. There are some reports that elevated insulin levels/hyperinsulinemia alone can precipitate insulin resistance and obesity, thus making the lower insulin levels in the Met + HFD PN of potential benefit (33, 34).

In the setting of the hyperinsulinemic euglycemic clamp, we found evidence of hepatic, cardiac, and adipose insulin resistance, including impaired suppression of lipolysis and glucose production in male HFD PN offspring. In this experiment, there was no difference in glucose infusion rates, indicating that increased hepatic glucose output in the HFD PN male offspring could be offset by increased peripheral glucose turnover. However, there was no evidence of increased glucose turnover in the tissues assessed here. It is possible that this increased glucose turnover is occurring in a tissue that was not analyzed during the clamp, for example, the brain. The increased whole-body glucose turnover rate at the baseline prior to insulin stimulation would also support this possibility, as brain glucose uptake is largely insulin independent (35). Other groups have shown the brain to be highly susceptible to programming in response to maternal HFD (32, 36). In a porcine model with high-fat diet given in the preconception period through lactation offspring had increased brain glucose uptake as newborns (37). However, this subsided at 1 month of age. The main effect of the metformin co-treatment seems to have been on the liver with no evidence of rescue of the muscle or adipose insulin resistance. However, in the Met + HFD PN group, clamp suppression of lipolysis became similar to Ctrl PN. Metformin is detected at higher levels in the portal circulation, making a liver-predominant effect in the offspring possible, despite having very low circulating pup metformin levels in our other lactational metformin models (20).

Our pilot gene set enrichment analysis identified upregulated PPAR-alpha pathway signaling in the livers of HFD PN male offspring fed ND. PPAR-alpha pathway responses are thought to be beneficial for the liver. Previous studies have shown that whole body and hepatic *Ppara* knockout animals are prone to developing hepatic steatosis (38). Indeed, PPAR-alpha agonists are being investigated as therapeutic options in NAFLD and metabolic syndrome. There could be two explanations for the PPAR-alpha findings in this study. First, this could represent a stress response that would be beneficial and may be protective for the HFD PN offspring livers. Further studies would be needed to identify the effects of antagonizing the PPAR-alpha in this experimental system. Another explanation is that the members of the PPAR-alpha pathway we found to be altered are not the major contributing members, and that these genes are exerting their effects on the offspring liver independently of PPAR-alpha signaling. Other studies have linked increased *Cpt1a, Pdk4*, and *Fgf21* with resolution of NASH in human liver biopsies (39), and we did not detect changes in these targets on the initial transcriptomics study. This study also showed that increased *Ppara* expression associated with NASH resolution, which could be pertinent to the findings in our HFD + MetPN group where liver *Ppara* expression was increased.

Our studies revealed an upregulation of Promyelocytic leukemia zinc finger (PLZF), also known as *Zbtb16*, in male HFD PN mice during the lactational exposure, at P16 and into adulthood. Increased *Zbtb16* gene expression has been observed in multiple models of diabetes, including db/db, streptozotocin (STZ) treated, and HFD-fed mice. Adenovirus-mediated overexpression of *Zbtb16* led to increased hepatic gluconeogenic gene expression, hyperinsulinemia, and insulin resistance in mice (40). In our model, it is possible that changes in the level of *Zbtb16* contributed to the increased hepatic glucose production and insulin resistance phenotypes in HFD PN offspring. *Zbtb16* expression was reduced in livers of HFD + Met PN males. This may represent a potential rescue mechanism provided by lactational metformin exposure, given that we also observed a tendency to improve hepatic glucose production in HFD + Met PN offspring. Further studies are needed to determine if post-natal metformin exposure reduces *Zbtb16* expression in HFD PN male offspring subjected to HFD rechallenge.

Cytochrome P450 omega-hydroxylase 4A14 (*Cyp4a14*) was also upregulated in the livers of HFD PN male mice. *Cyp4a14* is a direct transcriptional target of PPARa and is involved in fatty acid metabolism. Cyp4a family members metabolize arachidonic acid to generate pro-inflammatory mediator 20-hydroxyeicosatetraenoic acid (20-HETE), which is known to activate NFκB (41). In the context of liver disease, genetic ablation of *Cyp4a14* in mice protects against HFD-induced hepatic steatosis and methionine-choline-deficient (MCD)-diet-induced fibrosis and inflammation (42). Pharmacologic inhibition of Cyp4a family members decreased insulin resistance in HFD-fed mice (43). Dysregulation of *Cyp4a14* may contribute to the increased hepatic steatosis in HFD PN male offspring observed in the current study and to previously reported insulin resistance in HFD rechallenged HFD PN male mice (8).

In the pilot transcriptomics data and the “second-hit” male livers, we detected decreased levels of Cytochrome P450 Family 7 Subfamily A Member 1 (Cyp7a1). *Cyp7a1* encodes the rate-limiting enzyme that catalyzes the conversion of cholesterol to 7α-hydroxycholesterol in the classic bile acid synthesis pathway, playing a critical role in cholesterol and bile acid homeostasis (44). It has been shown that *Cyp7a1*^−/−^ mice fed a MCD diet to cause NAFLD have increased hepatic-free cholesterol compared to wild-type mice (45). Free cholesterol may accumulate in hepatic stellate cells and exacerbate liver fibrosis in NASH (46). Additionally, adenovirus-mediated *Cyp7a1* gene transduction has been shown to inhibit lipopolysaccharide (LPS)-induced p65 binding to NF-κB binding sites on cytokine gene promoters, possibly mediated by the activation of FXR via bile acids (45).

The enhanced hepatic steatosis and fibrosis observed in HFD PN + HFD male offspring were accompanied by an increased expression of *Mcp-1* and a decreased expression of *Il-6*. An increase in *Mcp-1* expression would suggest enhanced macrophage infiltration into the liver. However, when liver sections were analyzed for the presence of Mac2^+^ macrophages, we did not observe any difference in infiltration between Ctrl PN + HFD and HFD PN + HFD groups. This could be better assessed using flow cytometry. Within the liver, Kupffer cells and hepatic stellate cells also express the MCP-1 receptor, *Ccr2*. Expression of CCR2 on these cells may also contribute to the development of fibrosis after liver injury; however, this was not shown to be dependent on MCP-1(47). IL-6 is known to play both pro-inflammatory and anti-inflammatory roles in different contexts. In the liver, antibody blockade of IL-6 signaling led to enhanced hepatic steatosis in response to MCD diet (48). Conversely, chronic treatment of ob/ob mice with subcutaneous injection of recombinant IL-6 reduced hepatic steatosis and triglyceride levels (49). Taken together, the expression patterns that we observed in HFD PN + HFD male livers may be contributing pathological changes.

The effects we detected on the liver and insulin sensitivity are sexually dimorphic with the male offspring being predominantly impacted and the female offspring relatively spared. We previously reported the lack of effect of lactational HFD on female offspring insulin sensitivity and adiposity detected in our studies out to age 6 months (8). Male sex confers an increased risk of NAFLD in a clinical setting, and, in our mouse experimental results, we also detected this difference in outcomes. While both male and female offspring are heavier as a result of lactational HFD during the suckling period, when we study the offspring as young adults, there were no evident metabolic phenotypes in females. Perhaps, the effect of pubertal hormone exposure is protective in females. Indeed, *Cyp4a14* has sexually dimorphic expression in mice with increased levels in females. The decreased expression in males was caused by male-specific growth hormone secretory patterns and androgens (50). This may indicate a different role for this enzyme in female mice or different sensitivity to elevated levels in males. Finally, it is possible that the female mice have a milder phenotype that may be unmasked by a longer HFD rechallenge period or the addition of a stressor, like pregnancy.

In summary, these studies have provided evidence of a complex offspring response to lactational HFD exposures that leads to hepatic insulin resistance and increased risk of NAFLD in adult male offspring. Male HFD PN offspring also have evidence of insulin resistance in the heart and adipose tissue. We have identified potential molecular candidates that could be associated with the liver abnormalities observed in this model as they have been shown to cause insulin resistance, increased gluconeogenesis, hepatic steatosis, and fibrosis in other models. The unique aspects of this study are that a brief, indirect neonatal exposure to HFD may contribute to genetic perturbations in adulthood, leading to lifelong metabolic disease risk. Future studies will be designed to understand the impacts of these specific genes on the liver across the life span and how changes in the maternal milk that results from HFD exposure can regulate expression of these genes, possibly through epigenetic changes. Finally, we will also determine if the co-exposure of dams to HFD and metformin during lactation is able to rescue the offspring from the detrimental effects of an HFD “second-hit” stressor.

## Data Availability Statement

The datasets presented in this study can be found in online repositories. The names of the repository/repositories and accession number(s) can be found below: https://www.ncbi.nlm.nih.gov/geo/query/acc.cgi?acc=GSE182071.

## Ethics Statement

The animal study was reviewed and approved by University of Michigan Institutional Animal Care and Use Committee.

## Author Contributions

BG was responsible for the study design and conceived the original idea. BG, HH, MM, ZC, HS, MW, and NQ carried out the experiments. BG, HH, MM, ZC, NQ, and DB performed data analysis. Manuscript preparation was done by BG, HH, MM, ZC, PH, HS, and DB. Manuscript editing was completed by HH, MM, ZC, NQ, DB, and BG. Input and approval of the final manuscript were provided by all the authors.

## Funding

BG was supported by K08 DK102526 and R56 DK121787. DB was supported by R01 DK107535. Amino acid and long-chain fatty acid levels were analyzed at the Michigan Regional Comprehensive Metabolomics Resource Core (U24DK097153). Hyperinsulinemic euglycemic clamp experiments were done by the Mouse Metabolic Phenotyping Center (U2CDK110768). Aspects of this work were also planned in consultation with Core Directors from the Michigan Diabetes Research Center (DK020572 and DK089503). Histology was performed by the Rogel Cancer Center Histology Core (P30 CA04659229). RNA Sequencing was performed by the University of Michigan Advanced Genomics Core. ZC was funded by an internship through the Momentum Center of the University of Michigan. MM was supported by a University of Michigan Rackham Merit Fellowship.

## Conflict of Interest

The authors declare that the research was conducted in the absence of any commercial or financial relationships that could be construed as a potential conflict of interest.

## Publisher's Note

All claims expressed in this article are solely those of the authors and do not necessarily represent those of their affiliated organizations, or those of the publisher, the editors and the reviewers. Any product that may be evaluated in this article, or claim that may be made by its manufacturer, is not guaranteed or endorsed by the publisher.
