# Supplementary material for: Lactational High Fat Diet in Mice Causes Insulin Resistance and NAFLD in Male Offspring Which Is Partially Rescued by Maternal Metformin Treatment
Source: Front Nutr. 2021 Dec 15;8:759690. doi: 10.3389/fnut.2021.759690 (PMC8714922; doi:10.3389/fnut.2021.759690)
Supplement: Supplementary file 1 [file Data_Sheet_1.PDF]

Supplementary Material

1.1 Supplementary Figures

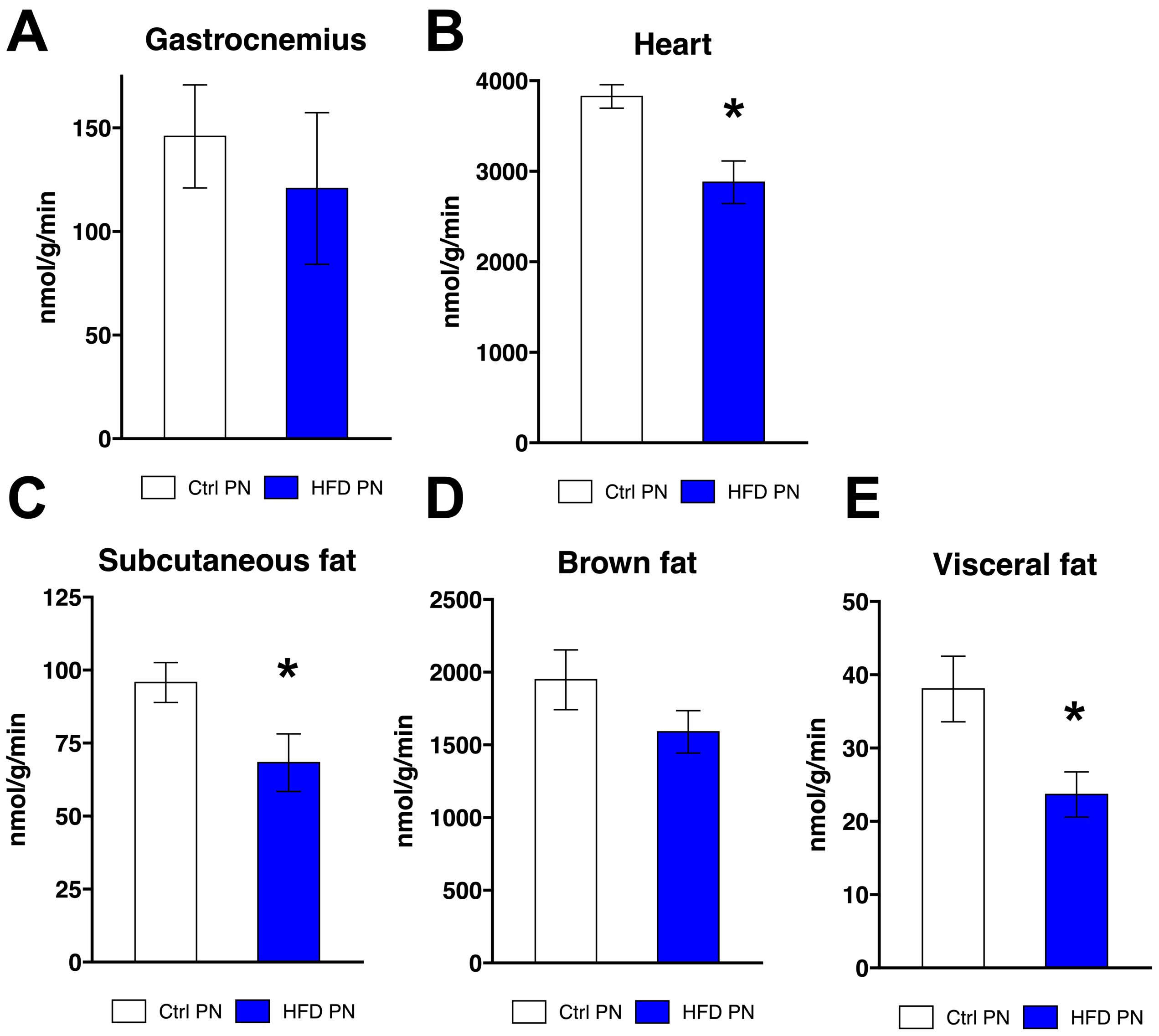

Supplementary Figure 1. Tissue glucose uptake during the hyperinsulinemic euglycemic clamp in adult male offspring at 12 weeks of age. Glucose uptake in gastrocnemius (A) heart (B) subcutaneous fat (C) brown fat (D) and visceral fat (E). N=9-11. White bars indicate control offspring and blue bars are HFD offspring. Ctrl=control, HFD=high fat diet, PN=postnatal. *$p<0.05$

Group	Summary
P16 ctrl	looks normal no steatosis
P16 ctrl	looks normal no steatosis
P16 ctrl	looks normal no steatosis
P16 HFD	looks normal no steatosis
P16 HFD	looks normal no steatosis
P16 HFD	looks normal no steatosis
P16 HFD	looks normal no steatosis
P16 HFD	looks normal no steatosis
P16 HFD	looks normal no steatosis
Ctrl PN	looks normal
Ctrl PN	looks normal
Ctrl PN	looks normal
Ctrl PN	a few dead hepactocytes, unclear significance; difficult to evaluate d/t staining variation
Ctrl PN	a few dead hepactocytes, unclear significance; difficult to evaluate d/t staining variation
Ctrl PN	a few dead hepactocytes, unclear significance; difficult to evaluate d/t staining variation
Ctrl PN	a few dead hepactocytes, unclear significance; difficult to evaluate d/t staining variation
Ctrl PN	a few dead hepactocytes, unclear significance; difficult to evaluate d/t staining variation
HFD PN	looks normal stain quality poor
HFD PN	looks normal stain quality poor
HFD PN	looks normal stain quality poor
HFD PN	looks normal stain quality poor
HFD PN	looks normal
HFD PN	looks normal
HFD PN	area of Zone 2 necrosis and neutrophils with coagulative necrosis of hepatocytes
HFD PN	looks normal stain quality poor
Ctrl PN+HFD	50% microvesicular steatosis, zone 3, <33% macrovesicular steatosis, no inflammation
Ctrl PN+HFD	50% microvesicular steatosis, zone 3, no macrovesicular steatosis, no inflammation
Ctrl PN+HFD	50% microvesicular steatosis, zone 3, no macrovesicular steatosis, no inflammation
Ctrl PN+HFD	80% microvesicular steatosis, zone 3, <33% macrovesicular steatosis, no inflammation
Ctrl PN+HFD	looks normal
Ctrl PN+HFD	looks normal
Ctrl PN+HFD	looks normal
Ctrl PN+HFD	tissue too shredded to evaluate; looks like 33% macrovescualr steatosis
HFD PN+HFD	80% microvesicular steatosis, zone 3, <33% macrovesicular steatosis,
HFD PN+HFD	50% microvesicular steatosis, zone 3, no macrovesicular steatosis, no inflammation
HFD PN+HFD	50% microvesicular steatosis, zone 3, no macrovesicular steatosis, no inflammation
HFD PN+HFD	80% microvesicular steatosis, zone 3, <33% macrovesicular steatosis, no inflammation
HFD PN+HFD	80% microvesicular steatosis, zone 3, <33% macrovesicular steatosis, no inflammation
HFD PN+HFD	80% microvesicular steatosis, zone 3, <33% macrovesicular steatosis, no inflammation
HFD PN+HFD	80% microvesicular steatosis, zone 3, <33% macrovesicular steatosis, no inflammation
HFD PN+HFD	80% microvesicular steatosis, zone 3, <33% macrovesicular steatosis, no inflammation

Supplementary Table 1. Pathologist scoring of hematoxylin & eosin (H&E) stained liver sections. The top group is from P16 liver. The middle section is from adult male liver on normal diet. The bottom portion is male liver after “second-hit” HFD re-challenge.
